# Molecular Heterogeneity in Pediatric Malignant Rhabdoid Tumors in Patients With Multi-Organ Involvement

**DOI:** 10.3389/fonc.2022.932337

**Published:** 2022-07-13

**Authors:** Katherine E. Miller, Gregory Wheeler, Stephanie LaHaye, Kathleen M. Schieffer, Sydney Cearlock, Lakshmi Prakruthi Rao Venkata, Alejandro Otero Bravo, Olivia E. Grischow, Benjamin J. Kelly, Peter White, Christopher R. Pierson, Daniel R. Boué, Selene C. Koo, Darren Klawinski, Mark A. Ranalli, Ammar Shaikhouni, Ralph Salloum, Margaret Shatara, Jeffrey R. Leonard, Richard K. Wilson, Catherine E. Cottrell, Elaine R. Mardis, Daniel C. Koboldt

**Affiliations:** ^1^ The Steve and Cindy Rasmussen Institute for Genomic Medicine, Abigail Wexner Research Institute at Nationwide Children’s Hospital, Columbus, OH, United States; ^2^ Department of Pediatrics, The Ohio State University College of Medicine, Columbus, OH, United States; ^3^ Department of Pathology, The Ohio State University College of Medicine, Columbus, OH, United States; ^4^ Department of Pathology and Laboratory Medicine, Nationwide Children’s Hospital, Columbus, OH, United States; ^5^ Department of Biomedical Education and Anatomy, Division of Anatomy, The Ohio State University College of Medicine, Columbus, OH, United States; ^6^ Department of Pathology, St. Jude Children’s Research Hospital, Memphis, TN, United States; ^7^ Division of Hematology, Oncology, and Bone Marrow Transplant, Nationwide Children’s Hospital, Columbus, OH, United States; ^8^ Pediatric Neuro-Oncology Program, Nationwide Children’s Hospital, Columbus, OH, United States; ^9^ Department of Neurosurgery, Nationwide Children’s Hospital, Columbus, OH, United States; ^10^ Department of Neurosurgery, The Ohio State University College of Medicine, Columbus, OH, United States; ^11^ The Division of Hematology and Oncology, St. Louis Children’s Hospital, Washington University School of Medicine, St. Louis, MO, United States

**Keywords:** atypical teratoid/rhabdoid tumor (AT/RT), malignant rhabdoid tumor (MRT), SMARCB1, next-generation sequencing, DNA methylation array

## Abstract

Rhabdoid tumors (RTs) of the brain (atypical teratoid/rhabdoid tumor; AT/RT) and extracranial sites (most often the kidney; RTK) are malignant tumors predominantly occurring in children, frequently those with *SMARCB1* germline alterations. Here we present data from seven RTs from three pediatric patients who all had multi-organ involvement. The tumors were analyzed using a multimodal molecular approach, which included exome sequencing of tumor and germline comparator and RNA sequencing and DNA array-based methylation profiling of tumors. *SMARCB1* germline alterations were identified in all patients and in all tumors. We observed a second hit in *SMARCB1 via* chr22 loss of heterozygosity. By methylation profiling, all tumors were classified as rhabdoid tumors with a corresponding subclassification within the MYC, TYR, or SHH AT/RT subgroups. Using RNA-seq gene expression clustering, we recapitulated the classification of known AT/RT subgroups. Synchronous brain and kidney tumors from the same patient showed different patterns of either copy number variants, single-nucleotide variants, and/or genome-wide DNA methylation, suggestive of non-clonal origin. Furthermore, we demonstrated that a lung and abdominal metastasis from two patients shared overlapping molecular features with the patient’s primary kidney tumor, indicating the likely origin of the metastasis. In addition to the *SMARCB1* events, we identified other whole-chromosome events and single-nucleotide variants in tumors, but none were found to be prognostic, diagnostic, or offer therapeutic potential for rhabdoid tumors. While our findings are of biological interest, there may also be clinical value in comprehensive molecular profiling in patients with multiple rhabdoid tumors, particularly given the potential prognostic and therapeutic implications for different rhabdoid tumor subgroups demonstrated in recent clinical trials and other large cohort studies.

## Introduction

Rhabdoid tumors (RT) are rare, malignant tumors diagnosed most often in early childhood. RTs are classified according to their anatomical location and most often arise in the brain (atypical teratoid/rhabdoid tumor; AT/RT) and/or extracranially, usually in the kidney (RTK) and sometimes in other soft tissues like muscles (1, 2). In the United States, AT/RT represents 10% of primary brain and central nervous system (CNS) tumors diagnosed in individuals less than 1 year of age (1). RTKs account for 18% of all renal tumors diagnosed in infants (2). Because of the rarity and aggressiveness of RTs and given the young age of many patients, there is no defined standard of care, and RTs remain one of the most lethal childhood tumors with overall survival rates <50% (3–5).

Almost all cases of RT, regardless of anatomical site, are molecularly characterized by the biallelic alteration of *SMARCB1*, leading to complete inactivation of the gene and, more rarely, inactivation of *SMARCA4* in a germline or somatic setting (6–8). *SMARCB1*, also called *INI1/BAF47/SNF5*, is an established tumor suppressor at 22q11.2 that encodes a subunit protein of the SWI/SNF chromatin remodeling complex (9). In approximately 35% of individuals diagnosed with an AT/RT or RTK, one of the *SMARCB1* alterations is present in the germline, predisposing the individual to the development of rhabdoid tumors (10, 11).

Despite the commonality of being driven by *SMARCB1* or *SMARCA4* loss, there is molecular and clinical heterogeneity among AT/RT. There are three known DNA methylation subgroups associated with AT/RT, referred to as TYR (characterized by the overexpression of melanosomal genes), SHH (characterized by the overexpression of sonic hedgehog signaling pathways), and MYC (characterized by the overexpression of both MYC proto-oncogene and HOX cluster genes) (12–14). AT/RT-TYR are diagnosed in the youngest population group (median age of diagnosis: 12 months) and usually arise in an infratentorial location (75%). AT/RT-SHH are predominantly supratentorial in location (65%) and are diagnosed in individuals with a median age of 20 months. AT/RT-MYC arise in the supratentorial region (50%), the infratentorial region (38%), and even extracranially in the spinal cord (12%) and represent the oldest population group within AT/RT diagnoses at a median age of 27 months. Extracranial rhabdoid tumors most often show a similarity with the AT/RT-MYC subgroup at the DNA methylation level (15, 16). The commonalities and overlap between subgroups of AT/RT and subgroups of extracranial RT (specifically, RTK) are not well characterized, particularly in patients who present with both types of tumors or in longitudinal samples from patients who experience metastasis of their primary tumor.

Here we present a case series of three pediatric patients diagnosed and treated for AT/RT and/or RTK. We analyzed multiple different tumors from each patient, totaling seven tumor samples, including primary tumors and metastases. Each tumor was comprehensively analyzed using a multimodal molecular characterization approach that included exome sequencing for detection of germline and somatic variants and copy number alterations, whole transcriptome sequencing (RNA-seq), DNA array-based methylation analyses, and clonality analysis. Our approach uncovered a consistent biallelic inactivation of *SMARCB1* as expected but revealed molecular heterogeneity among tumors from the same individual.

## Methods

### Human Subjects

Written informed consent was obtained for all participants in this study under a research protocol approved by the Institutional Review Board at Nationwide Children’s Hospital (IRB17-00206). We enrolled three individuals with a diagnosis of rhabdoid tumor. Our report includes three females: “patient 1”, “patient 2”, and “patient 3” diagnosed at age 10, 2, and 2 months, respectively. Two patients presented with multifocal synchronous AT/RT and RTK primary tumors, one of whom eventually had metastasis to the lungs. The third patient had a primary RTK and ultimately experienced tumor metastasis to the abdomen after an initial surgical resection. Snap-frozen disease-involved tissue was studied, when possible, but for some specimens only formalin-fixed, paraffin-embedded (FFPE) disease-involved tissue was available for study. A detailed description of the clinical history of each patient can be found in the supplementary file (**Case Descriptions**).

### Samples and Extractions

Normal comparator tissue was obtained from blood-derived peripheral blood mononuclear cells or from non-tumor kidney tissue in one individual. The tumor samples were obtained as either fresh-frozen or FFPE tissue and were used for the co-extraction of DNA (AllPrep DNA kit, Qiagen) and RNA (mirVana isolation kit, Thermo Fisher Scientific for frozen and High Pure isolation kit, Roche Life Science for FFPE tissues).

### Exome Sequencing and Analysis

Sequencing libraries were prepared for exome sequencing using NEBNext Ultra II FS DNA library prep kit (New England BioLabs). First, target enrichment by hybrid capture was performed by combining xGen Exome Research Panel with the xGenCNV Backbone and Cancer-Enriched Panels-Tech Access (Integrated DNA Technologies). The xGen exome panel (catalog number 10005153) targets 19,433 genes with a total probe coverage encompassing 39 Mb of genomic space, while the xGenCNV backbone panel (catalog number 1080569) consists of 9,115 individually synthesized probes combined with an additional 1,855 probes enriching cancer-associated gene regions. Libraries were then generated using the NEBNext Ultra II FS kit, and paired-end 151-bp reads were generated on NovaSeq6000. Alignment to human reference genome build GRCh38 and secondary analysis were performed using our previously published pipeline (17).

Germline variants were called using GATK’s HaplotypeCaller. The variants were then filtered based on the following characteristics: gnomAD population frequency <0.0001, depth of sequencing ≥8 reads, variant within protein-coding region or within 3 base pairs of canonical splice site, and presence of the gene within a previously published cancer predisposition list of 565 genes (18). VarScan2 and GATK were used to assess copy number variants (CNVs) and to detect loss of heterozygosity across all chromosomes (19). Copy number variation plots were generated from Varscan2-called segment files and plotted by centering around a zero-point determined by the median log-2 value of the segments of the first five chromosomes. Invariant copy data based on log-2 ratios were plotted as 100-bp windows in blue, wherein copy number variant segments were plotted as a red line by corresponding log-2 copy ratio and position. Loss of heterozygosity (LOH) data were plotted for tumor samples using the position (X axis) and variant allele frequency (VAF; Y axis) of called alleles filtered by a list of known biallelic sites. Points falling outside of an expected normal range of 25 to 75% were marked as LOH-supporting.

Somatic variants were called using MuTect2 (20). Somatic nonsynonymous SNVs and small insertions or deletions (indels) were filtered for quality (site quality ≥100), population frequency (gnomAD population frequency <0.0001), absence in the germline comparator sample, somatic alternate allele read depth (≥4 reads), minimum tumor VAF ≥5%, and gene location within a coding or splice site (≤3 base pairs) region. Variants passing all the aforementioned filters were manually reviewed in Integrated Genomics Viewer and then analyzed for the presence of the variant within a previously defined cancer hotspot (21) or the presence of the gene within a previously published cancer predisposition list of 565 genes (18). VarScan2 and GATK were used to assess CNVs and loss of heterozygosity across all chromosomes (19). Copy number variation and LOH data plots were generated as described for germline CNVs.

### DNA Array-Based Methylation Profiling

For each tumor studied, 250–500 ng of input DNA was bisulfite-converted (catalog number D5006, Zymo Research, Irvine, CA, USA) and, if applicable, treated using the Illumina FFPE restoration process (catalog number WG-321-1002, Illumina, San Diego, CA, USA). Bisulfite-converted DNAs, including methylated human DNA controls (catalog number D5014, Zymo Research, Irving, CA, USA), were hybridized to the Infinium Methylation EPIC BeadChip (catalog number WG-317-1001, Illumina, San Diego, CA, USA) following the Illumina Infinium HD Methylation protocol. Beadchips were imaged on the Illumina iScan System, and the resulting raw IDAT files were processed through a local installation of the German Cancer Research Center (DKFZ) DNA Methylation Brain Tumor Classifier, version 11b4 or 11b6 (22, 23). Uniform manifold approximation and projection (UMAP) plots were generated to assess the unsupervised clustering of the studied AT/RT samples, where only the most differentially methylated probes were considered. For comparison of our study samples with external DKFZ embryonal tumor samples (*i*.*e*., AT/RT, medulloblastomas, and embryonal tumors with multilayered rosettes), standard deviation ≥0.25 was used, which included 30,549 most differentially methylated probes for clustering analyses.

### RNA Sequencing and Gene Expression Analysis

Tumor RNA was subjected to DNase treatment and ribodepletion prior to library construction using NEBNext Ultra II Directional RNA library prep kit for Illumina (New England BioLabs). Paired-end 151-bp reads were generated on Illumina HiSeq 4000 and aligned to the human genome reference sequence build GRCh38. Alignment was performed using a custom in-house pipeline and the splice-aware aligner STAR (24). Clustering was performed by principal component analysis (PCA) on log10(*x* + 1) and quantile-normalized DESeq2 expression values using a panel of 36 genes with known relevance to AT/RT subtyping for MYC (*HOTAIR*, *HOXC4/5/6/8/9/10/11/12/13/AS1/AS5*, and *MYC*), SHH (*ASCL1*, *BOC*, *CDH6*, *DLL1/3*, *DTX1*, *GLI2*, *HES1/5/6*, *MYCN*, and *PTCH1*), and TYR (*BMP4*, *DCT*, *DNAH11*, *FGFR2*, *JAK1*, *MITF*, *OTX2*, *PDGFRB*, *SPEF1*, *TYR*, and *VEGFA*) groups (13).

### Clonality Analysis

We used superFreq with default parameters to determine clonality (25). superFreq uses exome BAM files from tumor samples and identifies tumor-specific single-nucleotide variants, indels, and copy number variants to track clones across multiple samples from the same patient.

## Results

### Tissue Pathology

The pathology review typically estimated a high tumor cellularity/content (average, 95%; range, 90–100%) and a wide range of necrosis (average, 12%; range, 0–40%) for all tumors studied (**Table 1**). As described in the clinical summaries (**Supplementary File- Case Descriptions**), the histologic findings in all tumors were determined by board-certified pathologists and were consistent with either AT/RT or extracranial RT supported by loss of INI1 staining.

**Table 1:** Summary of molecular findings.

ID	Comparatortissue	Germline*SMARCB1*	Tumorsite	Tumorcontent	Necrosis	CNS family (methylation score)	CNS AT/RT class (methylation score)
Patient 1	Blood	p.Pro215Leufs*14	Brain	95%	1%	AT/RT (0.9997)^a^	TYR (0.9997)^a^
			Kidney	90%	30%	AT/RT (0.9421)^a^	MYC (0.9078)^a^
			Lung (metastatic)	90%	40%	AT/RT (0.9998)^b^	MYC (0.9977)^b^
Patient 2	Kidney, non-tumor	1.88 Mb deletion at 22q11.22-q11.23	Brain	100%	0%	AT/RT (0.9980)^a^	SHH (0.9958)^a^
			Kidney	98%	5%	AT/RT (0.9971)^a^	MYC (0.9967)^a^
Patient 3	Blood	1.34 Mb deletion at 22q11.22-q11.23	Kidney	100%	0%	AT/RT (0.9997)^a^	MYC (0.9997)^a^
			Abdomen (metastatic)	90%	10%	AT/RT (0.9932)^b^	MYC (0.9823)^b^

Unless otherwise indicated, all tissue specimens were from a primary tumor. The estimates of tumor content and necrosis are based on a pathology review.

AT/RT, atypical teratoid rhabdoid tumor; MYC, MYC gene subgroup of AT/RT; SHH, sonic hedgehog subgroup of AT/RT; TYR, tyrosinase subgroup of AT/RT.

a Classifier versions used for the Heidelberg Brain Tumor and Sarcoma Classifiers: v11b4 CNS classifier.

b Classifier versions used for the Heidelberg Brain Tumor and Sarcoma Classifiers: v11b6 CNS classifier.

### Genomic Analysis

The average exome sequencing coverage depth for the tumor samples was 241X (range: 181X–289X) and for the germline comparator samples was 216X (range: 186X–266X). For all samples, an average of 97.9% of coding bases was covered by at least 20 reads (range, 97.0–98.6%) (**Supplementary Table S1**).

#### Germline Analysis

All three patients had a pathogenic germline alteration affecting *SMARCB1* identified by exome sequencing (**Table 1**). Patient 1 had a heterozygous frameshift variant in *SMARCB1* (p.Pro215Leufs*14), while patients 2 and 3 had a large deletion (>1 Mb) of chr22q11, a region which includes the *SMARCB1* gene, present in germline comparator tissue, thus confirming a diagnosis of rhabdoid tumor predisposition syndrome for all three.

#### Somatic Analysis

We observed a second somatic hit in *SMARCB1* in all tumor specimens *via* LOH of chr22, inclusive of the *SMARCB1* gene region. In patient 1, although heterozygous in the germline (48% VAF), the p.Pro215Leufs*14 variant exhibited much higher allele frequencies in all three tumor specimens (87% in primary brain, 68% in primary kidney, and 82% in lung metastasis) because of extensive copy-neutral LOH across chromosome 22q in all three tumor specimens (**Supplementary Figures S1A–C**). In patient 2, the chr22 deletion appeared homozygous in the patient’s primary brain and primary kidney tumors due to copy-neutral LOH across the entirety of chromosome 22 (**Supplementary Figures S1D, E**). We made similar observations in patient 3, who harbored a slightly smaller germline deletion on chr22 (1.34 Mb) that appeared homozygous in both primary kidney and metastatic abdominal tumors due to copy-neutral LOH affecting the entire chromosome (**Supplementary Figures S1F, G**).

### DNA Methylation Profiling and Gene Expression Clustering and CNV Analysis Reveal Heterogeneity Between Tumors From the Same Patient

We generated DNA methylation profiles using Illumina EPIC 850K microarray for our cohort of tumors and analyzed the data using the DKFZ brain tumor methylation classifier v11b4 or v11b6, which provides the classification of 184 known central nervous system tumors (22). All tumors, including intra- and extracranial tumors, matched most closely with the methylation family of AT/RT, and all had confidence scores >0.90 (range: 0.9421–0.9997; **Table 1**). The brain tumor methylation classifier also assigns samples to different methylation subgroups or classes within the AT/RT family. In our cohort, one brain sample was predicted to be a TYR subgroup and one was predicted to be a SHH subgroup, while the other five samples (all non-CNS) were predicted to be MYC subgroups. Synchronous tumors from the same patient (patients 1 and 2) demonstrated epigenetic heterogeneity and were assigned different methylation subgroups, as visualized by the appearance in distinct UMAP clusters alongside other embryonal tumors from the DKFZ database (**Supplementary Figure S2**). The primary RTK and abdominal metastatic tumors from patient 3 were both classified as MYC and clustered together.

We also performed RNA-seq on all tumors, yielding >80,000,000 total reads for each RNA sample (**Supplementary Table S1**). To explore the heterogeneity of gene expression between the subgroups of RTs, we performed PCA of the RNA-seq data using 36 genes (see “*Methods*”) known to be divergently expressed in specific AT/RT subgroups, which include TYR/MITF/others for TYR subgroup, NOTCH signaling genes (*e*.*g*., *ASCL1*, *HES5/6*, and *DLL1/3*) and SHH signaling genes (*e*.*g*., *MYCN* and *GLI2*) for SHH subgroup, and *MYC/HOTAIR/HOXC* cluster genes for MYC subgroup (13). Similar to our methylation clustering, synchronous brain and extracranial tumors from the same patient demonstrated a variation in expression profiles and appeared in distinct clusters (**Supplementary Figure S3**). The resultant gene expression clustering data supported the subgroup classification predicted by methylation analysis for each sample.

### Clonal Tracking Using Single-Nucleotide Variants and Copy Number Variations Reveals Heterogeneity Between Tumors From the Same Patient

#### Patient 1

**Figure 1:**
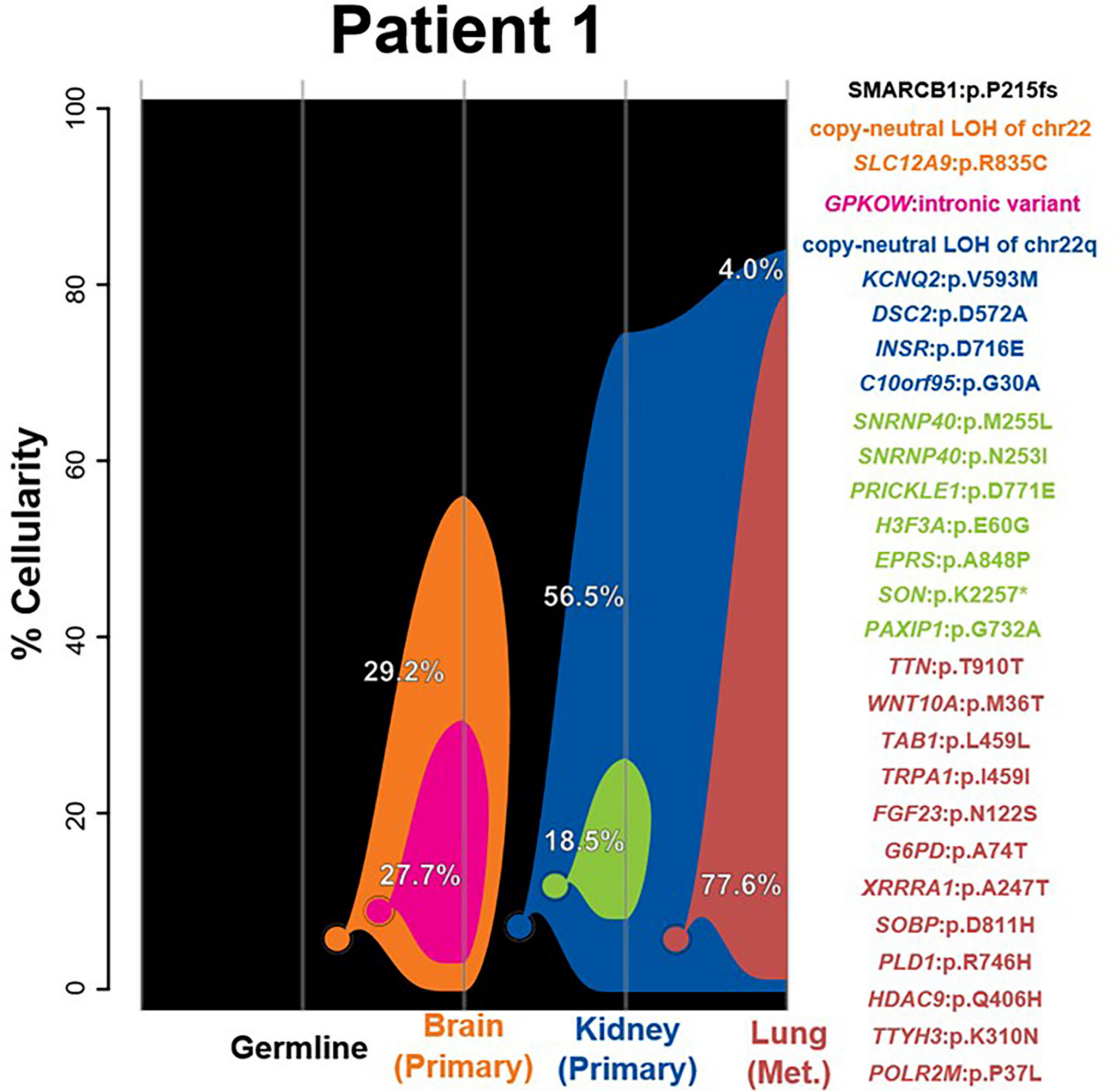
**–3**Clonality analysis. River plots showing the composition of clones in multiple tumor samples plus matched normal blood (germline) sample. Vertical lines indicate a sample, labeled on the X-axis. Colored circles indicate the origins of a clone from a single cell, either of the germline (black) or a preceding clone. Colored outlines show the cellularity of clones (Y-axis) in each sample. The percentages shown (in white or black text) indicate the cellularity of a tumor clone in each sample that was identified, excluding the cells of any descendant clones. Clone percentages sum up to the total tumor cellularity of a sample. The samples are arranged to most clearly visualize clonal descent but do not represent a formal time-series. The copy number variants, loss-of-heterozygosity events, and single-nucleotide variants identified in the tumor samples as well as relevant germline predisposition variants are listed on the right and colored by which clone they belong to.

While all samples possess second-hit somatic events resulting in the loss of any functional *SMARCB1* allele, the brain lesion demonstrated a whole-chromosome loss of chr22, whereas the two extracranial samples exhibited LOH of chr22q only. Clonality analysis differentiated the brain and extracranial lesions into two distinct tumorigenic origins on the basis of a number of other variants. Of the 25 SNVs included in clonal clustering, none was found to be shared between the brain and extracranial tumors (**Figure 1** and **Supplementary Table S2**). The kidney and lung tumors were found to be closely related, with the kidney and lung tumors sharing four SNVs. Like the patient’s kidney tumor, the lung metastasis was also classified as MYC subgroup based on methylation profiling, further supporting the probable shared clonal origin of these two extracranial tumors. Each extracranial sample was found to possess a uniquely derived subclone, with kidney and lung samples gaining a number of exclusive SNVs (7 and 12, respectively). The subclone identified in the brain sample was differentiated only by one SNV, an intronic variant in *GPKOW* (**Supplementary Table S2**). No other CNVs besides chr22 or chr22q LOH were identified in any of the tumors (**Supplementary Figures S1A–C**). Despite the presence of two independent tumor lineages and as many as five distinct clones, no new diagnostic, prognostic, or therapeutically applicable SNVs or CNVs were found in any clones beyond the *SMARCB1* events.

#### Patient 2

**Figure 2:**
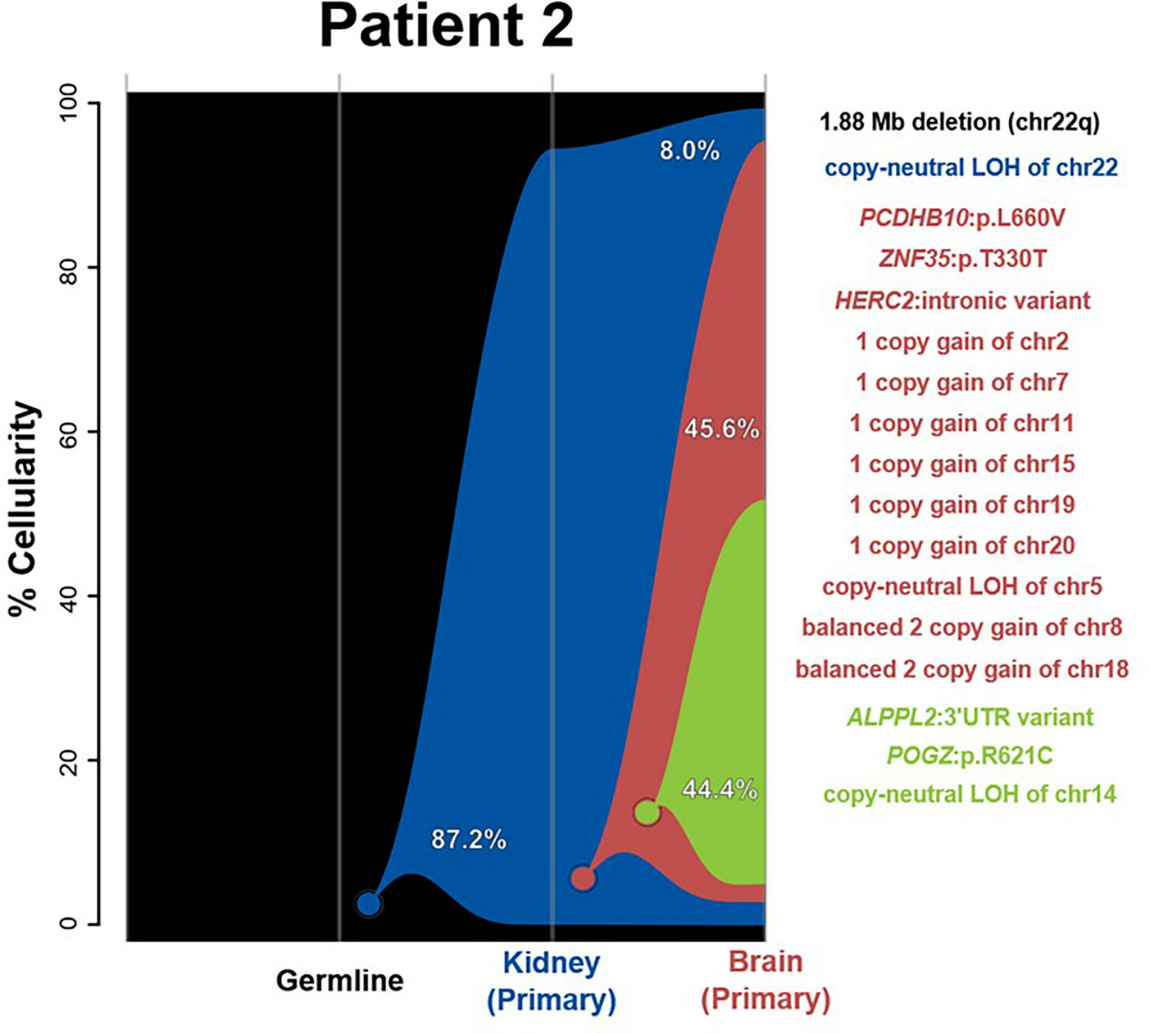
River plots show the composition of clones in multiple tumor samples plus matched normal blood (germline) sample. Vertical lines indicate a sample, labeled on the X-axis. Colored circles indicate the origins of a clone from a single cell, either of the germline (black) or a preceding clone. Colored outlines show the cellularity of clones (Y-axis) in each sample. Percentages shown (in white or black text) indicate the cellularity of a tumor clone in each sample that was identified, excluding the cells of any descendant clones. Clone percentages sum to the total tumor cellularity of a sample. Samples are arranged to most clearly visualize clonal descent but do not represent a formal time-series. Copy number variants (CNVs), loss-of-heterozygosity events (LOH), and single nucleotide variants (SNVs) identified in the tumor samples, as well as relevant germline predisposition variants, are listed on the right and colored by which clone they belong to.

Clonality analysis indicated a shared origin for kidney and brain samples as the most parsimonious clonal history for this case. However, only a single event—whole-chromosome copy-neutral LOH of chr22—is shared between the two tumors. While the kidney lesion was not found to have any SNVs or CNVs beyond the initial chr22 LOH, the copy number analysis of the brain tumor revealed several chromosomal aberrations and two distinct sequentially derived clones (**Figure 2**). The first clone (red) added three SNVs and nine full-chromosome CNV events—a single-copy gain of chr2, chr7, chr11, chr15, chr19, and chr20; balanced two-copy gains of chr8 and chr18; and a copy-neutral LOH of chr5 (**Figure 2** and **Supplementary Figures 1D,E**). The second, further-derived clone (green) added an additional of two SNVs plus a loss of heterozygosity of chr14. These observed molecular differences between the brain and kidney tumors is further supported by their classification into different methylation subgroups (SHH and MYC, respectively), indicating that the tumors do not share a clonal origin. Aside from chr22 LOH, none of the SNVs or CNVs unique to the brain lesion was found to be meaningful in terms of prognosis, diagnosis, or therapeutic potential for AT/RT (26).

#### Patient 3

**Figure 3:**
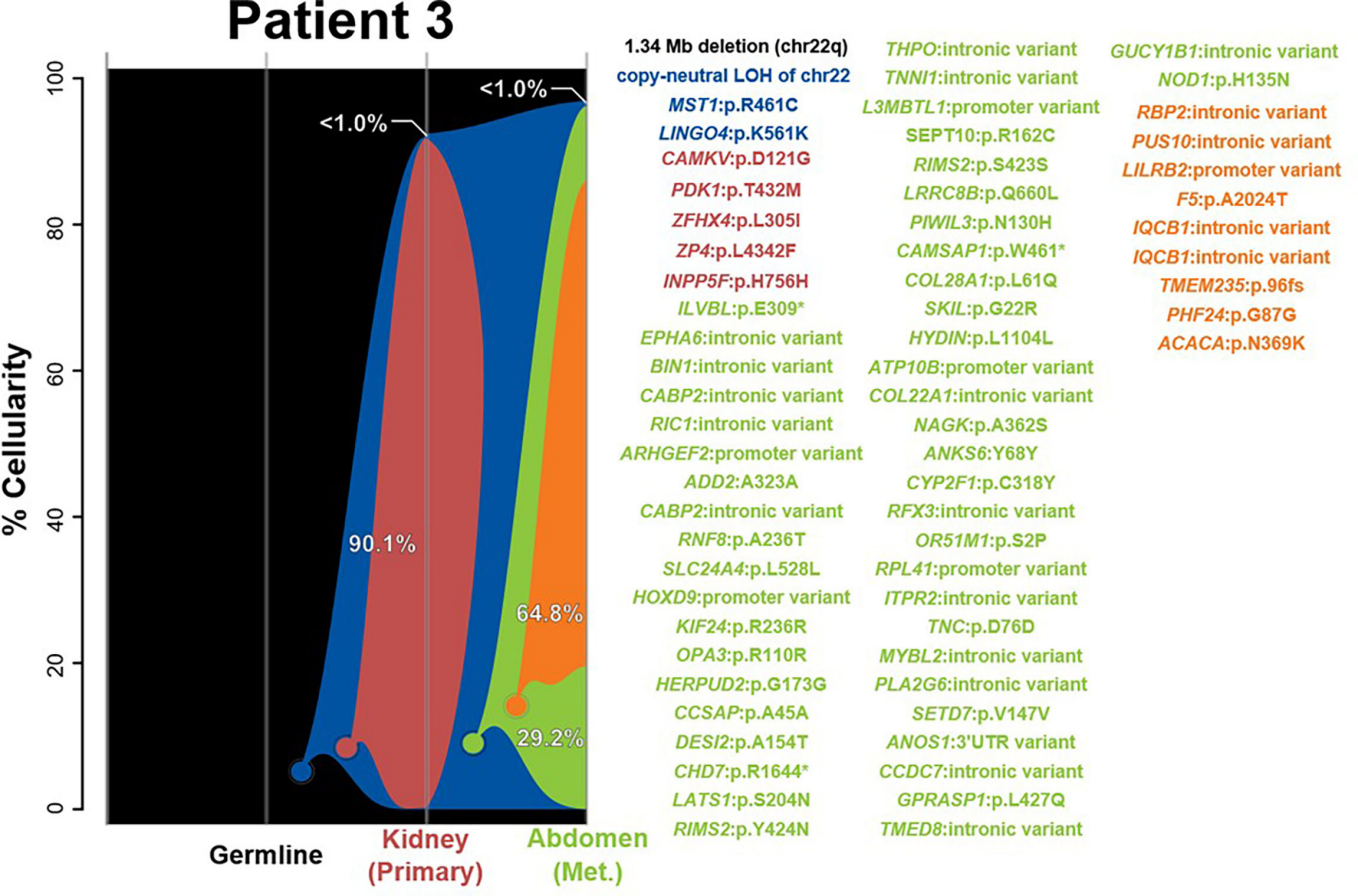
River plots show the composition of clones in multiple tumor samples plus matched normal blood (germline) sample. Vertical lines indicate a sample, labeled on the X-axis. Colored circles indicate the origins of a clone from a single cell, either of the germline (black) or a preceding clone. Colored outlines show the cellularity of clones (Y-axis) in each sample. Percentages shown (in white or black text) indicate the cellularity of a tumor clone in each sample that was identified, excluding the cells of any descendant clones. Clone percentages sum to the total tumor cellularity of a sample. Samples are arranged to most clearly visualize clonal descent but do not represent a formal time-series. Copy number variants (CNVs), loss-of-heterozygosity events (LOH), and single nucleotide variants (SNVs) identified in the tumor samples, as well as relevant germline predisposition variants, are listed on the right and colored by which clone they belong to.

The two samples in this case, of the kidney and abdomen, were determined to share a single tumorigenic origin on the basis of shared SNVs in *MST1* and *LINGO4*, in addition to the chr22 LOH event often observed in rhabdoid tumors (**Figure 3** and **Supplementary Figures 1F, G**). Methylation profiling classified both as MYC, supporting the abdominal metastases that share similar epigenetic features to the kidney tumor and therefore may share a clonal origin. From this shared clonal origin, three additional distinct subclones have arisen. The one subclone present in the kidney (red) sample possesses five additional SNVs, while the two sequentially derived abdominal subclones add 49 (green) and 9 (orange) SNVs, respectively. In each sample, the derived clones have nearly entirely replaced the ancestral cell population, resulting in the two tumors being substantially genetically distinct in a large number of high-frequency variants; none of these derived variants, however, was found to be informative of prognosis, diagnosis, or therapeutic potential for rhabdoid tumors.

## Discussion

We analyzed seven tumor tissue samples from three pediatric patients diagnosed with rhabdoid tumors of the brain and/or extracranial sites and aimed to systematically assess each individual case using a multimodal approach of DNA and RNA sequencing plus methylation profiling. As expected, all tumors exhibited biallelic inactivation of *SMARCB1*, including a germline *SMARCB1* alteration in every patient. We also analyzed tumors using DNA array-based methylation and RNAseq-based gene expression analyses to characterize tumors based on known molecular subgroups of AT/RT (12). Our use of the v11b4/b6 brain tumor methylation classifier provided confident classifications for tumors as either MYC, SHH, or TYR AT/RT subgroups, even for extracranial RTs (22). We confirmed the use of analyzing gene expression data from RNA-seq as an orthogonal method for identifying distinct molecular AT/RT subgroups by evaluating the expression of known subgroup-specific marker genes (13).

Our integrated analysis of synchronous tumors from the same patient revealed several interesting findings. First, RTs originating in and outside the brain in each case showed molecularly heterogeneous methylation profiling and were classified as different subgroups (patient 1 brain = TYR and kidney = MYC; patient 2 brain = SHH and kidney = MYC). While the finding of divergent methylation patterns in synchronous rhabdoid tumors has been reported before, our integrated analyses using exome sequencing and clonal tracking demonstrated that, aside from chr22 LOH, the brain and extracranial tumors demonstrated further molecular heterogeneity and never shared any tumor SNVs or CNVs (**Figures 1**, **2**) (15, 27). Although the tumors from patient 2 were initially predicted to share a clonal origin (using superFreq software), this is likely because of the commonality of chr22 LOH and the fact that the kidney tumor did not possess any other CNVs or SNVs. In fact, LOH of chr22 or chr22q was a ubiquitous event observed in all tumors within our cohort and is likely the most common mechanism for a second hit in *SMARCB1* in patients with rhabdoid tumor predisposition syndrome (*i*.*e*., germline *SMARCB1* mutation). As chr22 LOH is common in this tumor type, it is most likely that the true explanation is that an indistinguishable event occurred in two separate instances for the brain and extracranial RT to have been formed—for example, in patient 2. This explanation is supported by the high cellularity of the first derived brain clone (red) in patient 2, suggesting that it may be the true tumorigenic ancestor of the brain lesion (**Figure 2**). Due to the lack of any other shared genomic alterations (besides chr22 LOH) and the lack of a biological mechanism for metastasis between the kidney and brain, the more plausible explanation is that the loss of chr22 has occurred twice separately. However, as the events alone are indistinguishable, a metastatic event is conceivably possible and cannot be excluded with certainty. In patient 3, we observed the same methylation profile for both kidney and abdominal lesions, and additionally we identified two shared SNVs between the two tumors at >40% VAF, indicating a likely shared clonal origin (**Supplementary Table S2**). While most of the tumors in our cohort appeared to derive clones, it was composed of either non-coding SNVs (intronic or promoter regions) or other passenger mutations, primarily nonsynonymous variants in genes not previously associated with cancer. Interestingly, only one tumor in our cohort (patient 2 brain) had additional CNVs besides chr22 LOH, highlighting the importance of using exome sequencing to study the tumors fully.

Given that AT/RT is a heterogenous disease with different subgroups, the goal is that molecular studies and findings can be used to guide therapy and improve patient outcomes. Several epidemiological studies have independently reported that patients with AT/RT-TYR or *ASCL1-*expressing (NOTCH signaling) ATRT-SHH tumors have a better prognosis, but more analyses on larger prospective cohorts are needed to confirm these findings (12, 28–30). In addition, several preclinical studies have identified drugs and drug-like inhibitors with different therapeutic effects in molecular subgroups of AT/RTs (31–33). Therefore, it is likely that molecular subgrouping of rhabdoid tumors is expected to affect patient management in the future, as there may be differences in response to different therapies and overall survival. While our findings are of biological interest, there may also be clinical value in using comprehensive molecular profiling to diagnose and classify rhabdoid synchronous tumors, particularly given the potential prognostic and therapeutic implications for different rhabdoid tumor subgroups.

## Data Availability Statement

DNA and RNA sequencing data for this study has been deposited to dbGaP under accession number phs001820.v1.p1. dbGaP identifiers for each sample are listed in **Supplementary Table 1**.

## Ethics Statement

The studies involving human participants were reviewed and approved by Institutional Review Board at Nationwide Children’s Hospital. Written informed consent to participate in this study was provided by the participants’ legal guardian/next of kin. Written informed consent was obtained from the minor(s)’ legal guardian/next of kin for the publication of any potentially identifiable images or data included in this article.

## Author Contributions

KM: Conceptualization, Writing- Original Draft; GW: Formal analysis, Writing- Original Draft; SL: Formal analysis; KS: Formal analysis, Writing - Review and Editing; SC: Formal analysis, Writing- Original Draft; LV: Formal analysis; AB: Formal analysis; OG: Formal analysis; BK: Formal analysis; PW: Supervision; CP: Resources; DB: Resources, Writing - Review and Editing; SK: Resources; DK: Resources; MR: Resources; AS: Resources; RS: Resources; MS: Resources; JL: Resources; RW: Supervision; CC: Supervision, Writing - Review and Editing; EM: Conceptualization, Writing- Original Draft; DCK: Conceptualization, Writing- Original Draft. All authors contributed to the article and approved the submitted version.

## Funding

This work was supported by the Nationwide Foundation Pediatric Innovation Fund.

## Conflict of Interest

The authors declare that the research was conducted in the absence of any commercial or financial relationships that could be construed as a potential conflict of interest.

## Publisher’s Note

All claims expressed in this article are solely those of the authors and do not necessarily represent those of their affiliated organizations, or those of the publisher, the editors and the reviewers. Any product that may be evaluated in this article, or claim that may be made by its manufacturer, is not guaranteed or endorsed by the publisher.

## Supplementary Material

Supplementary Figure 1 Genome-wide copy number plots. In the top window of each panel are copy number plots, where blue points represent the log-2 ratio for the tumor relative to the normal specimen and red lines represent copy number variants segments as called by GATK. The heterozygosity plots (bottom window of each panel) show the tumor variant allele frequency (VAF) for heterozygous germline variants, which are colored red if they exhibit significant evidence of loss of heterozygosity (LOH) from the expected 50% VAF. The horizontal blue lines indicate contiguous LOH segments. Included are patient 1 primary brain tumor **(A)**, primary kidney tumor **(B)**, and metastatic lung tumor **(C)**; patient 2 primary brain tumor **(D)**, and primary kidney tumor **(E)**; and patient 3 primary kidney tumor **(F)** and metastatic abdominal tumor **(G)**.

Supplementary Figure 2 Unsupervised clustering by uniform manifold approximation and projection (UMAP) of embryonal tumors indicates the grouping of rhabdoid tumor (RT) tumors by methylation classification. The seven samples from our study were compared to 545 embryonal tumors [atypical teratoid (AT)/RT, medulloblastoma, and embryonal tumors with multilayered rosettes] described by Capper *et al.* (22) by unsupervised UMAP clustering, using the most differentially methylated probes (standard deviation ≥0.25, *n* = 30549 probes). The seven tumors from our cohort were grouped according to their predicted classifications, as assigned by the DKFZ CNS Classifier v11b4/v11b6. The samples from our cohort are outlined in black and are filled with color by their AT/RT subgroup as called by previous methylation profiling.

Supplementary Figure 3 Clustering by RNA-seq expression recapitulates subgrouping of rhabdoid tumors. Principal component analysis was performed by utilizing 36 genes (see “*Methods*”) known to be differentially expressed in distinct AT/RT subgroups. The samples are colored by their RT subgroup as called by methylation profiling and as central nervous system (CNS) or non-CNS indicative of the tumor location.

Supplementary Table 1 Exome sequencing metrics. Top: the 10 samples obtained from individuals are listed along with the type of tissue (tumor or comparator), sample type, mean depth of target exon coverage, and percent of targeted bases per gene sequenced to 20× coverage. Bottom: metrics from RNA-sequencing. The seven samples upon which RNA-seq was performed are listed along with tumor type, sample preservation type, total number of reads, number of reads mapped to GRCh38, and number of coding and untranslated region reads. The corresponding dbGap ID for each sample is listed. FFPE, formalin-fixed paraffin-embedded; GRCh38, human genome assembly (hg38) from Genome Reference Consortium; UTR, untranslated region

Supplementary Table 2 Single-nucleotide variants and copy number variants detected in clonality analysis. Variants detected in clonal tracking are listed with corresponding variant allele frequencies predicted from exome sequencing data. The color of the clone corresponding to –**5** is also noted.

CASE DESCRIPTIONS Clinical history of all three patients, which includes diagnostic details, treatment regimens, and the current clinical status of each patient.

## References

[1] Ostrom QT Chen Y P Ondracek A Farah P Gittleman H. The Descriptive Epidemiology of Atypical Teratoid/Rhabdoid Tumors in the United States, 2001-2010. Neuro Oncol (2014) 16(10):1392–9. doi: 10.1093/neuonc/nou090 PMC4165422 24847086

[2] Brennan B Stiller C Bourdeaut F. Extracranial Rhabdoid Tumours: What We Have Learned So Far and Future Directions. Lancet Oncol (2013) 14(8):e329–36. doi: 10.1016/S1470-2045(13)70088-3 23816299

[3] Shan Y Cai J Han Y Xie C Gao H Zhang L. An Analysis of the Diagnosis, Clinical Characteristics, Treatment, and Survival Outcomes of 36 Extracranial Malignant Rhabdoid Tumor Patients. Transl Pediatr (2021) 10(6):1598–609. doi: 10.21037/tp-20-459 PMC8261579 34295774

[4] Cai W Liu X Ge W Wu D Xu J Bai R. Factors Affecting the Outcomes of Patients With Malignant Rhabdoid Tumors: A Population-Based Study. Int J Med Sci (2021) 18(4):911–20. doi: 10.7150/ijms.51186 PMC7807195 33456348

[5] Pai Panandiker AS Merchant TE Beltran C Wu S Sharma S Boop FA. Sequencing of Local Therapy Affects the Pattern of Treatment Failure and Survival in Children With Atypical Teratoid Rhabdoid Tumors of the Central Nervous System. Int J Radiat Oncol Biol Phys (2012) 82(5):1756–63. doi: 10.1016/j.ijrobp.2011.02.059 PMC3530399 21601374

[6] Versteege I Sevenet N Lange J Rousseau-Merck MF Ambros P Handgretinger R. Truncating Mutations of Hsnf5/INI1 in Aggressive Paediatric Cancer. Nature (1998) 394(6689):203–6. doi: 10.1038/28212 9671307

[7] Hasselblatt M Nagel I Oyen F Bartelheim K Russell RB Schuller U. SMARCA4-Mutated Atypical Teratoid/Rhabdoid Tumors are Associated With Inherited Germline Alterations and Poor Prognosis. Acta Neuropathol (2014) 128(3):453–6. doi: 10.1007/s00401-014-1323-x 25060813

[8] Schneppenheim R Fruhwald MC Gesk S Hasselblatt M Jeibmann A Kordes U. Germline Nonsense Mutation and Somatic Inactivation of SMARCA4/BRG1 in a Family With Rhabdoid Tumor Predisposition Syndrome. Am J Hum Genet (2010) 86(2):279–84. doi: 10.1016/j.ajhg.2010.01.013 PMC2820190 20137775

[9] Kohashi K Oda Y. Oncogenic Roles of SMARCB1/INI1 and its Deficient Tumors. Cancer Sci (2017) 108(4):547–52. doi: 10.1111/cas.13173 PMC5406539 28109176

[10] Eaton KW Tooke LS Wainwright LM Judkins AR Biegel JA. Spectrum of SMARCB1/INI1 Mutations in Familial and Sporadic Rhabdoid Tumors. Pediatr Blood Cancer (2011) 56(1):7–15. doi: 10.1002/pbc.22831 21108436 PMC3086793

[11] Biegel JA Zhou JY Rorke LB Stenstrom C Wainwright LM Fogelgren B. Germ-Line and Acquired Mutations of INI1 in Atypical Teratoid and Rhabdoid Tumors. Cancer Res (1999) 59(1):74–9. 9892189

[12] Ho B Johann PD Grabovska Y De Dieu Andrianteranagna MJ Yao F Fruhwald M. Molecular Subgrouping of Atypical Teratoid/Rhabdoid Tumors-a Reinvestigation and Current Consensus. Neuro Oncol (2020) 22(5):613–24. doi: 10.1093/neuonc/noz235 PMC7229260 31889194

[13] Johann PD Erkek S Zapatka M Kerl K Buchhalter I Hovestadt V. Atypical Teratoid/Rhabdoid Tumors Are Comprised of Three Epigenetic Subgroups With Distinct Enhancer Landscapes. Cancer Cell (2016) 29(3):379–93. doi: 10.1016/j.ccell.2016.02.001 26923874

[14] Torchia J Golbourn B Feng S Ho KC Sin-Chan P Vasiljevic A. Integrated (Epi)-Genomic Analyses Identify Subgroup-Specific Therapeutic Targets in CNS Rhabdoid Tumors. Cancer Cell (2016) 30(6):891–908. doi: 10.1016/j.ccell.2016.11.003 27960086 PMC5500911

[15] Pinto EM Hamideh D Bahrami A Orr BA Lin T Pounds S. Malignant Rhabdoid Tumors Originating Within and Outside the Central Nervous System are Clinically and Molecularly Heterogeneous. Acta Neuropathol (2018) 136(2):315–26. doi: 10.1007/s00401-018-1814-2 PMC6063764 29428974

[16] Chun HE Johann PD Milne K Zapatka M Buellesbach A Ishaque N. Identification and Analyses of Extra-Cranial and Cranial Rhabdoid Tumor Molecular Subgroups Reveal Tumors With Cytotoxic T Cell Infiltration. Cell Rep (2019) 29(8):2338–54 e7. doi: 10.1016/j.celrep.2019.10.013 31708418 PMC6905433

[17] Kelly BJ Fitch JR Hu Y Corsmeier DJ Zhong H Wetzel AN. Churchill: An Ultra-Fast, Deterministic, Highly Scalable and Balanced Parallelization Strategy for the Discovery of Human Genetic Variation in Clinical and Population-Scale Genomics. Genome Biol (2015) 16:6. doi: 10.1186/s13059-014-0577-x 25600152 PMC4333267

[18] Zhang J Walsh MF Wu G Edmonson MN Gruber TA Easton J. Germline Mutations in Predisposition Genes in Pediatric Cancer. N Engl J Med (2015) 373(24):2336–46. doi: 10.1056/NEJMoa1508054 PMC4734119 26580448

[19] Koboldt DC Zhang Q Larson DE Shen D McLellan MD Lin L. VarScan 2: Somatic Mutation and Copy Number Alteration Discovery in Cancer by Exome Sequencing. Genome Res (2012) 22(3):568–76. doi: 10.1101/gr.129684.111 PMC3290792 22300766

[20] Cibulskis K Lawrence MS Carter SL Sivachenko A Jaffe D Sougnez C. Sensitive Detection of Somatic Point Mutations in Impure and Heterogeneous Cancer Samples. Nat Biotechnol (2013) 31(3):213–9. doi: 10.1038/nbt.2514 PMC3833702 23396013

[21] Chang MT Bhattarai TS Schram AM Bielski CM Donoghue MTA Jonsson P. Accelerating Discovery of Functional Mutant Alleles in Cancer. Cancer Discovery (2018) 8(2):174–83. doi: 10.1158/2159-8290.CD-17-0321 PMC5809279 29247016

[22] Capper D Jones DTW Sill M Hovestadt V Schrimpf D Sturm D. DNA Methylation-Based Classification of Central Nervous System Tumours. Nature (2018) 555(7697):469–74. doi: 10.1038/nature26000 PMC6093218 29539639

[23] Koelsche C Schrimpf D Stichel D Sill M Sahm F Reuss DE. Sarcoma Classification by DNA Methylation Profiling. Nat Commun (2021) 12(1):498. doi: 10.1038/s41467-020-20603-4 33479225 PMC7819999

[24] Dobin A Davis CA Schlesinger F Drenkow J Zaleski C Jha S. STAR: Ultrafast Universal RNA-Seq Aligner. Bioinformatics (2013) 29(1):15–21. doi: 10.1093/bioinformatics/bts635 23104886 PMC3530905

[25] Flensburg C Sargeant T Oshlack A Majewski IJ. SuperFreq: Integrated Mutation Detection and Clonal Tracking in Cancer. PloS Comput Biol (2020) 16(2):e1007603. doi: 10.1371/journal.pcbi.1007603 32053599 PMC7043783

[26] Mikhail FM Biegel JA Cooley LD Dubuc AM Hirsch B Horner VL. Technical Laboratory Standards for Interpretation and Reporting of Acquired Copy-Number Abnormalities and Copy-Neutral Loss of Heterozygosity in Neoplastic Disorders: A Joint Consensus Recommendation From the American College of Medical Genetics and Genomics (ACMG) and the Cancer Genomics Consortium (CGC). Genet Med (2019) 21(9):1903–16. doi: 10.1038/s41436-019-0545-7 31138931

[27] Thomas C Knerlich-Lukoschus F Reinhard H Johann PD Sturm D Sahm F. Two Molecularly Distinct Atypical Teratoid/Rhabdoid Tumors (or Tumor Components) Occurring in an Infant With Rhabdoid Tumor Predisposition Syndrome 1. Acta Neuropathol (2019) 137(5):847–50. doi: 10.1007/s00401-019-02001-3 30945057

[28] Fruhwald MC Hasselblatt M Nemes K Bens S Steinbugl M Johann PD. Age and DNA Methylation Subgroup as Potential Independent Risk Factors for Treatment Stratification in Children With Atypical Teratoid/Rhabdoid Tumors. Neuro Oncol (2020) 22(7):1006–17. doi: 10.1093/neuonc/noz244 PMC7339901 31883020

[29] Reddy AT Strother DR Judkins AR Burger PC Pollack IF Krailo MD. Efficacy of High-Dose Chemotherapy and Three-Dimensional Conformal Radiation for Atypical Teratoid/Rhabdoid Tumor: A Report From the Children's Oncology Group Trial Acns0333. J Clin Oncol (2020) 38(11):1175–85. doi: 10.1200/JCO.19.01776 PMC7145589 32105509

[30] Upadhyaya SA Robinson GW Onar-Thomas A Orr BA Johann P Wu G. Relevance of Molecular Groups in Children With Newly Diagnosed Atypical Teratoid Rhabdoid Tumor: Results From Prospective St. Jude Multi-Institutional Trials. Clin Cancer Res (2021) 27(10):2879–89. doi: 10.1158/1078-0432.CCR-20-4731 PMC8127412 33737307

[31] Meel MH Guillen Navarro M de Gooijer MC Metselaar DS Waranecki P Breur M. MEK/MELK Inhibition and Blood-Brain Barrier Deficiencies in Atypical Teratoid/Rhabdoid Tumors. Neuro Oncol (2020) 22(1):58–69. doi: 10.1093/neuonc/noz151 31504799 PMC6954444

[32] Wang SZ Poore B Alt J Price A Allen SJ Hanaford AR. Unbiased Metabolic Profiling Predicts Sensitivity of High MYC-Expressing Atypical Teratoid/Rhabdoid Tumors to Glutamine Inhibition With 6-Diazo-5-Oxo-L-Norleucine. Clin Cancer Res (2019) 25(19):5925–36. doi: 10.1158/1078-0432.CCR-19-0189 PMC6781869 31300448

[33] Alimova I Pierce A Danis E Donson A Birks DK Griesinger A. Inhibition of MYC Attenuates Tumor Cell Self-Renewal and Promotes Senescence in SMARCB1-Deficient Group 2 Atypical Teratoid Rhabdoid Tumors to Suppress Tumor Growth In Vivo. Int J Cancer (2019) 144(8):1983–95. doi: 10.1002/ijc.31873 30230537

