# Supplementary figures and images for: Molecular Heterogeneity in Pediatric Malignant Rhabdoid Tumors in Patients With Multi-Organ Involvement
Source: Front Oncol. 2022 Jul 13;12:932337. doi: 10.3389/fonc.2022.932337 (PMC9326117; doi:10.3389/fonc.2022.932337)

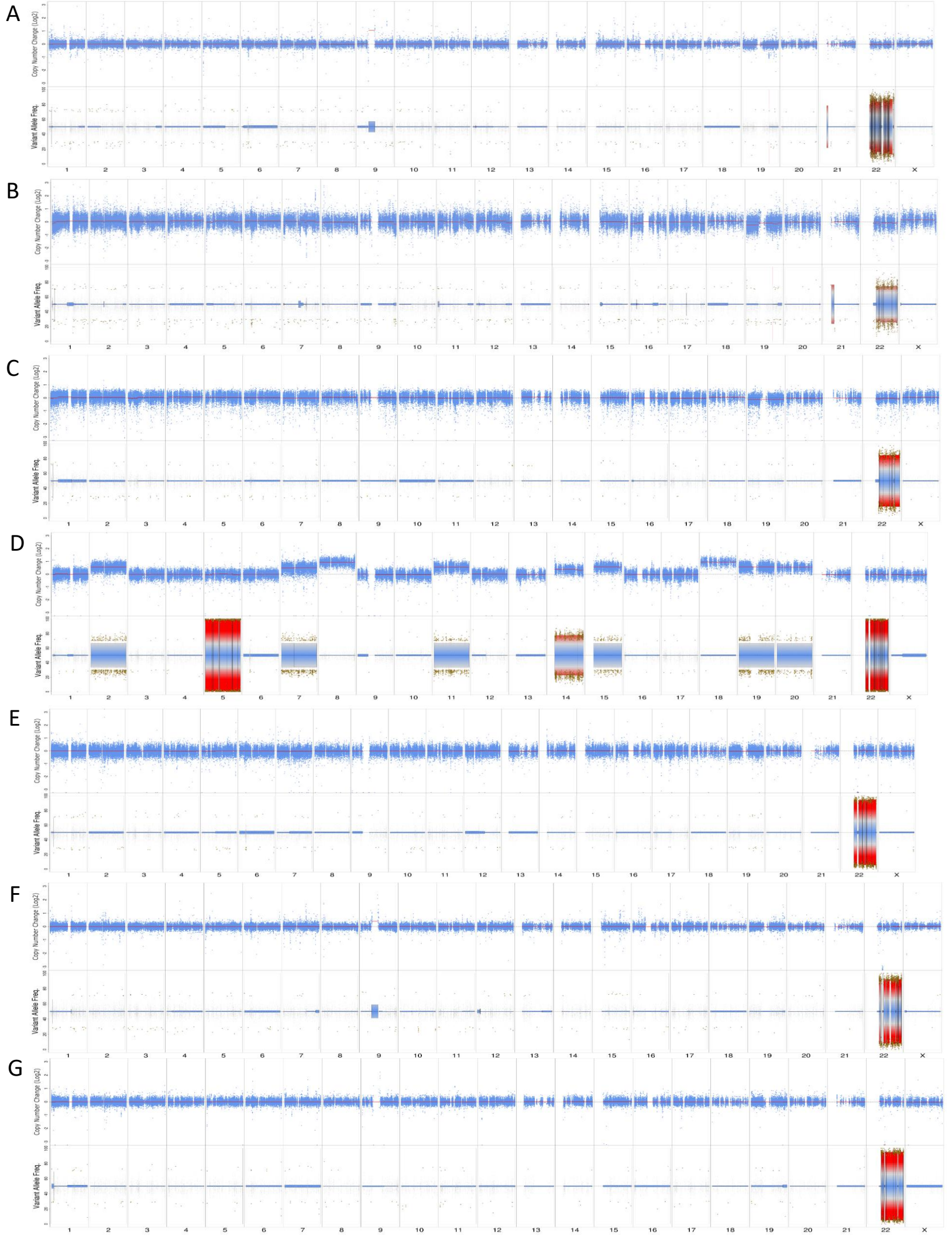

Supplement: Supplementary Figure 1 — Genome-wide copy number plots. In the top window of each panel are copy number plots, where blue points represent the log-2 ratio for the tumor relative to the normal specimen and red lines represent copy number variants segments as called by GATK. The heterozygosity plots (bottom window of each panel) show the tumor variant allele frequency (VAF) for heterozygous germline variants, which are colored red if they exhibit significant evidence of loss of heterozygosity (LOH) from the expected 50% VAF. The horizontal blue lines indicate contiguous LOH segments. Included are patient 1 primary brain tumor (A), primary kidney tumor (B), and metastatic lung tumor (C); patient 2 primary brain tumor (D), and primary kidney tumor (E); and patient 3 primary kidney tumor (F) and metastatic abdominal tumor (G). [file Image_1.pdf]

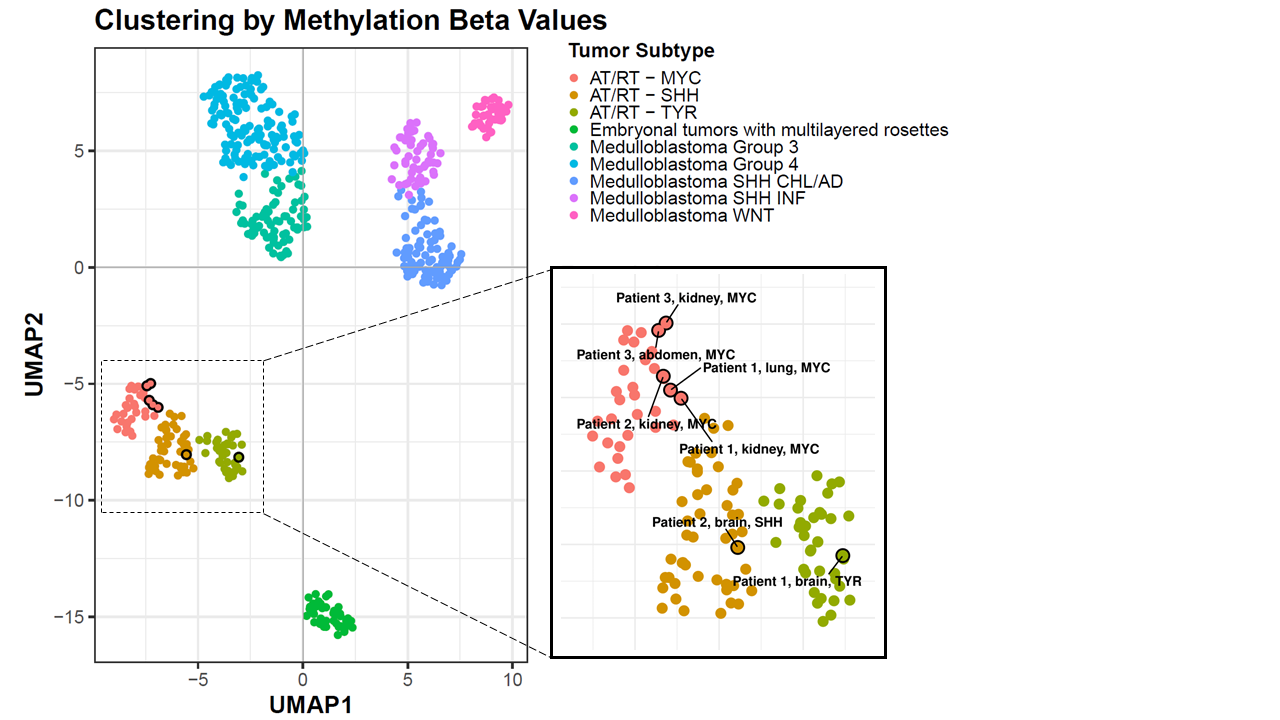

Supplement: Supplementary Figure 2 — Unsupervised clustering by uniform manifold approximation and projection (UMAP) of embryonal tumors indicates the grouping of rhabdoid tumor (RT) tumors by methylation classification. The seven samples from our study were compared to 545 embryonal tumors [atypical teratoid (AT)/RT, medulloblastoma, and embryonal tumors with multilayered rosettes] described by Capper et al. (22) by unsupervised UMAP clustering, using the most differentially methylated probes (standard deviation ≥0.25, n = 30549 probes). The seven tumors from our cohort were grouped according to their predicted classifications, as assigned by the DKFZ CNS Classifier v11b4/v11b6. The samples from our cohort are outlined in black and are filled with color by their AT/RT subgroup as called by previous methylation profiling. [file Image_2.jpeg]

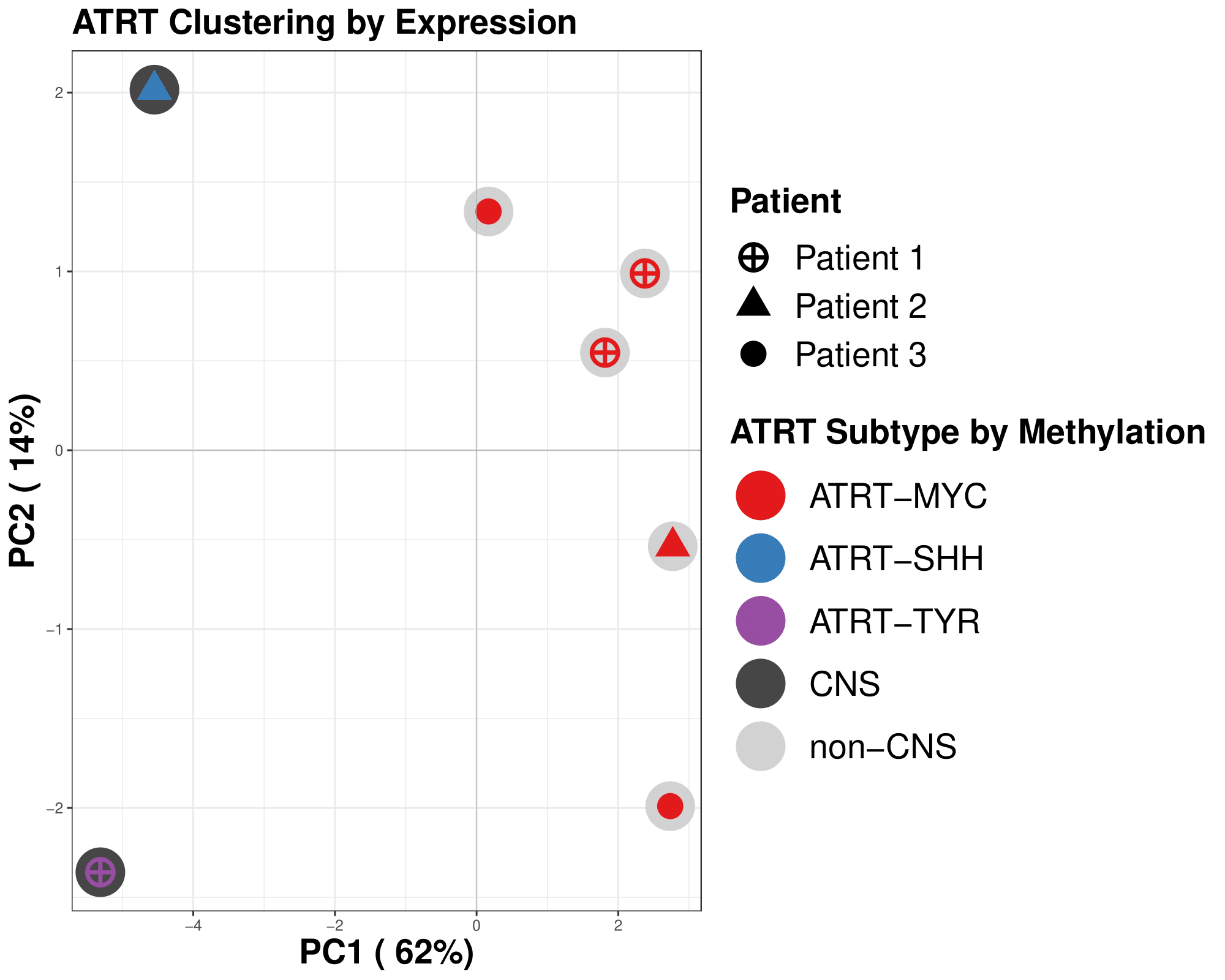

Supplement: Supplementary Figure 3 — Clustering by RNA-seq expression recapitulates subgrouping of rhabdoid tumors. Principal component analysis was performed by utilizing 36 genes (see “Methods”) known to be differentially expressed in distinct AT/RT subgroups. The samples are colored by their RT subgroup as called by methylation profiling and as central nervous system (CNS) or non-CNS indicative of the tumor location. [file Image_3.jpeg]
