# Supplementary material for: Molecular Heterogeneity in Pediatric Malignant Rhabdoid Tumors in Patients With Multi-Organ Involvement
Source: Front Oncol. 2022 Jul 13;12:932337. doi: 10.3389/fonc.2022.932337 (PMC9326117; doi:10.3389/fonc.2022.932337)
Supplement: CASE DESCRIPTIONS — Clinical history of all three patients, which includes diagnostic details, treatment regimens, and the current clinical status of each patient. [file DataSheet_1.docx]

**Molecular heterogeneity in pediatric malignant rhabdoid tumors**

**in patients with multi-organ involvement**

Katherine E. Miller^1,2^, Gregory Wheeler^1^, Stephanie LaHaye^1^, Kathleen M. Schieffer^1,3^, Sydney Cearlock^1^, Lakshmi Prakruthi Rao Venkata^1^, Alejandro Otero Bravo^1^, Olivia E. Grischow^1^, Benjamin J. Kelly^1^, Peter White^1,2^, Christopher R. Pierson^3,4,5^, Daniel R. Boué^3,4^, Selene C. Koo^3,4^, Diana Osorio^2,7,8^, Darren Klawinski^7,8^, Mark A. Ranalli^2,7^, Ammar Shaikhouni^9,10^, Ralph Salloum^2,7,8^, Margaret Shatara^7,8^, Jeffrey R. Leonard^2,9,10^, Catherine E. Cottrell^1,2,3^, Elaine R. Mardis^1,2,10^ & Daniel C. Koboldt^1,2^

^1^The Steve and Cindy Rasmussen Institute for Genomic Medicine, Abigail Wexner Research Institute at Nationwide Children's Hospital, Columbus, Ohio, USA

^2^Department of Pediatrics, The Ohio State University College of Medicine, Columbus, Ohio, USA

^3^Department of Pathology, The Ohio State University College of Medicine, Columbus, Ohio, USA

^4^Department of Pathology and Laboratory Medicine, Nationwide Children’s Hospital, Columbus, Ohio, USA

^5^Department of Biomedical Education & Anatomy, Division of Anatomy, The Ohio State University College of Medicine, Columbus, OH USA

^6^Department of Pathology, St. Jude Children’s Research Hospital, Memphis, Tennessee, USA

^7^Division of Hematology, Oncology, and Bone Marrow Transplant, Nationwide Children's Hospital, Columbus, Ohio, USA

^8^Pediatric Neuro-Oncology Program, Nationwide Children’s Hospital, Columbus, Ohio, USA

^9^Department of Neurosurgery, Nationwide Children’s Hospital, Columbus, Ohio, USA

^10^Department of Neurosurgery, The Ohio State University College of Medicine, Columbus, Ohio, USA

SUPPLEMENTARY DATA FILE- Case Descriptions

*Patient 1*

Patient 1 is a female who presented at 10 months of age to the emergency department due to parental concerns for progressive abdominal distention. A computed tomography (CT) scan revealed a left renal mass, and a nephrectomy was performed. Pathology was consistent with a rhabdoid tumor with renal sinus invasion with 9 of 10 lymph nodes testing positive for metastatic disease. Further evaluation with full body positron emission tomography (PET) imaging showed focal uptake at a mass in the left cerebello pontine angle. The brain mass was resected, and pathology was consistent with a diagnosis of AT/RT. INI1 nuclear immunostaining was lost in tumor cells. The patient began treatment with vincristine, cisplatin, doxorubicin, cyclophosphamide, and triple intrathecal chemotherapy with methotrexate, cytarabine, and hydrocortisone; this was followed by three tandem transplants with high dose chemotherapy with carboplatin and thiotepa and autologous stem cell rescue. Approximately four months after the completion of treatment she was found to have a metastatic lesion in the right lung, which was resected, and she was enrolled on an emergency investigational new drug protocol with Tazemetostat (ClinicalTrials.gov Identifier: NCT03213665). She experienced further pulmonary progression after approximately seven months and therapy was discontinued. She then enrolled on a phase 1 trial with palbociclib, cyclophosphamide, and topotecan. She developed right pulmonary and pleural-based nodules which were debulked. As she recovered from the procedure, she was enrolled in hospice and died from her disease, nearly two years after initial diagnosis.

*Patient 2*

Patient 2 is a female who presented to medical attention two months after birth with vomiting, decreased oral intake, and lethargy. She was noted to have bulging fontanelles and increased head circumference. A head ultrasound revealed hydrocephalus and a posterior fossa mass. She was admitted to the pediatric intensive care unit and underwent an MRI of the brain and spine which further delineated a posterior fossa mass centered in the fourth ventricle. A CT scan of the chest, abdomen, and pelvis was performed which also showed a large hypodense tumor within the right kidney centered in the interpolar region. Initially the patient had a gross total resection of the mass in the posterior fossa followed by a right radical nephroureterectomy approximately two weeks later. Pathological evaluation of the tumors revealed an AT/RT in the posterior fossa and an RT of the right kidney. INI1 immunohistochemical stain was lost in tumor cells. She received chemotherapy with vincristine, cisplatin, doxorubicin, cyclophosphamide, and triple intrathecal chemotherapy with methotrexate, cytarabine, and hydrocortisone; this was followed by two tandem transplants and high dose chemotherapy with carboplatin and thiotepa and autologous stem cell rescue. She is now off therapy for over 58 months without evidence of relapsed disease.

*Patient 3*

Patient 3 is a female who presented at two months of age with an abdominal mass and failure to thrive. She had an abdominal ultrasound followed by a CT of the abdomen which demonstrated a right retroperitoneal mass, and she subsequently underwent right radical nephroureterectomy. Pathology of the renal mass confirmed an RTK, and loss of INI1 expression was indicated by immunostaining. While a tectal mass (identified by full body PET scan) was thought to be AT/RT, no biopsy was performed. The patient began chemotherapy with vincristine, cisplatin, doxorubicin, cyclophosphamide, and triple intrathecal chemotherapy with methotrexate, cytarabine, and hydrocortisone; this was followed by three tandem transplants with high dose chemotherapy with carboplatin and thiotepa and autologous stem cell rescue. Approximately three months after completion of her third autologous transplant, an MRI of the abdomen revealed a new, peripherally enhancing retroperitoneal mass in the right lower quadrant. Exploratory laparotomy and biopsy revealed diffuse metastases throughout the abdominal cavity. The pathology of the retroperitoneal mass, as well as samples of the multiple abdominal tumors, was consistent with a recurrence of rhabdoid tumor. She was admitted to hospice and died two months after tumor recurrence.
